# Vagal Withdrawal and Susceptibility to Cardiac Arrhythmias in Rats with High Trait Aggressiveness

**DOI:** 10.1371/journal.pone.0068316

**Published:** 2013-07-04

**Authors:** Luca Carnevali, Mimosa Trombini, Alberto Porta, Nicola Montano, Sietse F. de Boer, Andrea Sgoifo

**Affiliations:** 1 Stress Physiology Lab, Department of Neuroscience, University of Parma, Parma, Italy; 2 Department of Biomedical Sciences for Health, Istituto Ortopedico Galeazzi, University of Milano, Milano, Italy; 3 Department of Biomedical and Clinical Sciences, Luigi Sacco Hospital, University of Milano, Milano, Italy; 4 ICRC-Department of Cardiovascular Diseases, St. Anne’s University Hospital, Brno, Czech Republic; 5 Department of Behavioural Physiology, Center for Behaviour and Neurosciences, University of Groningen, Groningen, The Netherlands; University of Adelaide, Australia

## Abstract

Personality characteristics, e.g. aggressiveness, have long been associated with an increased risk of cardiac disease. However, the underlying mechanisms remain unclear. In this study we used a rodent model for characterizing cardiac autonomic modulation in rats that differ widely in their level of aggressive behavior. To reach this goal, high-aggressive (HA, n = 10) and non-aggressive (NA, n = 10) rats were selected from a population (n = 121) of adult male Wild-type Groningen rats on the basis of their latency time to attack (ALT, s) a male intruder in a resident-intruder test lasting 600 s. In order to obtain information on their cardiac autonomic modulation, ECG recordings were subsequently obtained via radiotelemetry at rest, during stressful stimuli and under autonomic pharmacological manipulations, and analyzed by means of time- and frequency-domain indexes of heart rate variability. During resting conditions, HA rats (ALT<90 s) displayed reduced heart rate variability, mostly in terms of lower vagal modulation compared to NA rats (ALT>600 s). Exposure to stressful stimuli (i.e. restraint and psychosocial stress) provoked similar tachycardic responses between the two groups. However, under stress conditions HA rats displayed a reduced vagal antagonism and an increased incidence of tachyarrhythmias compared to NA rats. In addition, beta-adrenergic pharmacological stimulation induced a much larger incidence of ventricular tachyarrhythmias in HA rats compared to NA counterparts. These findings are consistent with the view that high levels of aggressive behavior in rats are associated to signs of cardiac autonomic impairment and increased arrhythmogenic susceptibility that may predict vulnerability to cardiac morbidity and mortality.

## Introduction

In humans, there are large individual differences in the susceptibility for cardiac disorders. The hypothesis that this inter-individual variability may be, in part, influenced by aspects of personality has been subject of debate and research for a long time. The groundbreaking work of Friedman and Rosenman in the late 1950 s still represents a hallmark on this regard [1]. They identified two types of behavior patterns (types A and B) that relate to individual stress responsivity and found that type A individuals (aggressive, hostile, impatient, competitive, achievement striving) were more vulnerable to heart disease than type B counterparts (relative absence of type A characteristics). Several failures to replicate this finding later questioned the role of the type A behavior pattern as a cardiac risk factor [2] [3]. This prompted researchers to examine individual components of the multifaceted type A behavior, as inconsistent association between the pattern and cardiac disease might indicate that only certain traits of type A personality influenced cardiac health. In particular, over the last twenty years a considerable number of studies has supported a relationship between the individual tendency to exhibit antagonistic interpersonal type A behaviors such us aggressiveness and hostility and increased risk for the onset and progression of cardiac disease [4]–[8].

Despite this, a mechanistic hypothesis has not yet been addressed conclusively in human studies. Putative pathophysiological mechanisms may include an impairment of the autonomic nervous system control over cardiac function. Abundant evidence demonstrates that reduced autonomic modulation of the heart, as shown by heart rate variability (HRV) measurements, predicts the development of heart disease in initially healthy subjects [9] [10], as well as poorer survival rate in patients with myocardial infarction [11] [12] or heart failure [13].

A deeper insight into the underlying mechanisms might be facilitated by the use of an objective and unbiased animal model since most described behavioral traits in humans are identifiable in animal models as well. Like humans, the way most animal species cope with stressful situations shows a high variability in behavioral responses. In feral rodent populations, a major feature of behavioral coping is the individual tendency to exhibit aggressive intraspecific behaviors [14]. In line with the characterization of personality in many other animal species [15]–[18], high levels of aggression in rodents are considered an important indicator and component of a more general proactive coping style, whereas low levels of aggression are believed to be a reflection of a reactive coping style [19] [20]. These divergent behavioral coping styles have frequently been associated with different patterns of both autonomic nervous and endocrine (re)activity [14] [19]. However, the investigation of the cardiac autonomic control of these distinct behavioral and physiological coping styles has been conducted only sporadically and provided inconclusive evidence [21] [22].

Given these considerations, in the current study we sought to characterize in detail the autonomic neural modulation of heart rate in two groups of male Wild-type Groningen rats (Rattus norvegicus) that differed largely in their level of aggressive behavior. This rat strain was chosen because, in contrast to other laboratory rat strains, it shows a wide and consistent individual variation in aggressive behavior [14]. The behavioral categorization was based on a trait-like characteristic, i.e. high-aggressive or non-aggressive towards a male unfamiliar conspecific intruder. This study included several key goals: (a) to assess sympathetic and parasympathetic influences on heart rate via time- and frequency-domain analysis of HRV at rest and during stress conditions, (b) to assess the relative contribution of vagal control over heart rate by means of cholinergic muscarinic blockade with methylscopolamine, (c) to quantify intrinsic heart rate under double pharmacological autonomic blockade, via beta-adrenoceptor and muscarinic receptor antagonists, and (d) to investigate susceptibility to cardiac arrhythmias under stress conditions and following β-receptor pharmacological stimulation. We tested the hypothesis that high levels of aggressive behavior in rats would be directly related to specific features of autonomic neural modulation of heart rate, which would justify the use of this rodent model in preclinical studies investigating the pathophysiological mechanisms linking aggressiveness and cardiac disease vulnerability.

## Methods

### Ethics Statement and Animals

Experimental procedures and protocols were approved by the Veterinarian Animal Care and Use Committee of Parma University, with animals cared for in accordance with the European Community Council Directives of 22 September 2010 (2010/63/UE).

In this study we used 4-month-old male Wild-type Groningen rats (*Rattus norvegicus*) weighing approximately 380 g. This rat population, originally derived from the University of Groningen (the Netherlands), is currently bred in our laboratory under conventional conditions, at ambient temperature of 22±2°C and on a reversed 12∶12 light-dark cycle (light on at 19∶00 h), with food and water available *ad libitium*.

### Preliminary Behavioral Testing for Aggressiveness

121 Wild-type rats were assessed for the display of aggressive behavior towards male unfamiliar conspecific intruders using a standard resident-intruder aggression test [23]. Individual experiments were carried out in 6 different cohorts of animals over a period of 16 months, using 20–21 animals in each experiment.

Ten days before the test, each rat was housed with a conspecific oviduct-ligated female partner to stimulate territorial behavior [24] [23]. Before the start of the test, the female partner was removed (approximately 15 min in advance) and an unfamiliar male Wistar rat was introduced into the home cage of the experimental rat. The intruder Wistar rats weighed on average 250 g (3 months old) and were socially housed. The test was repeated on three consecutive days, using every time a different intruder, in order to avoid familiarity between the opponents and obtain a reliable characterization of aggressive traits [14].

**Figure 1:**
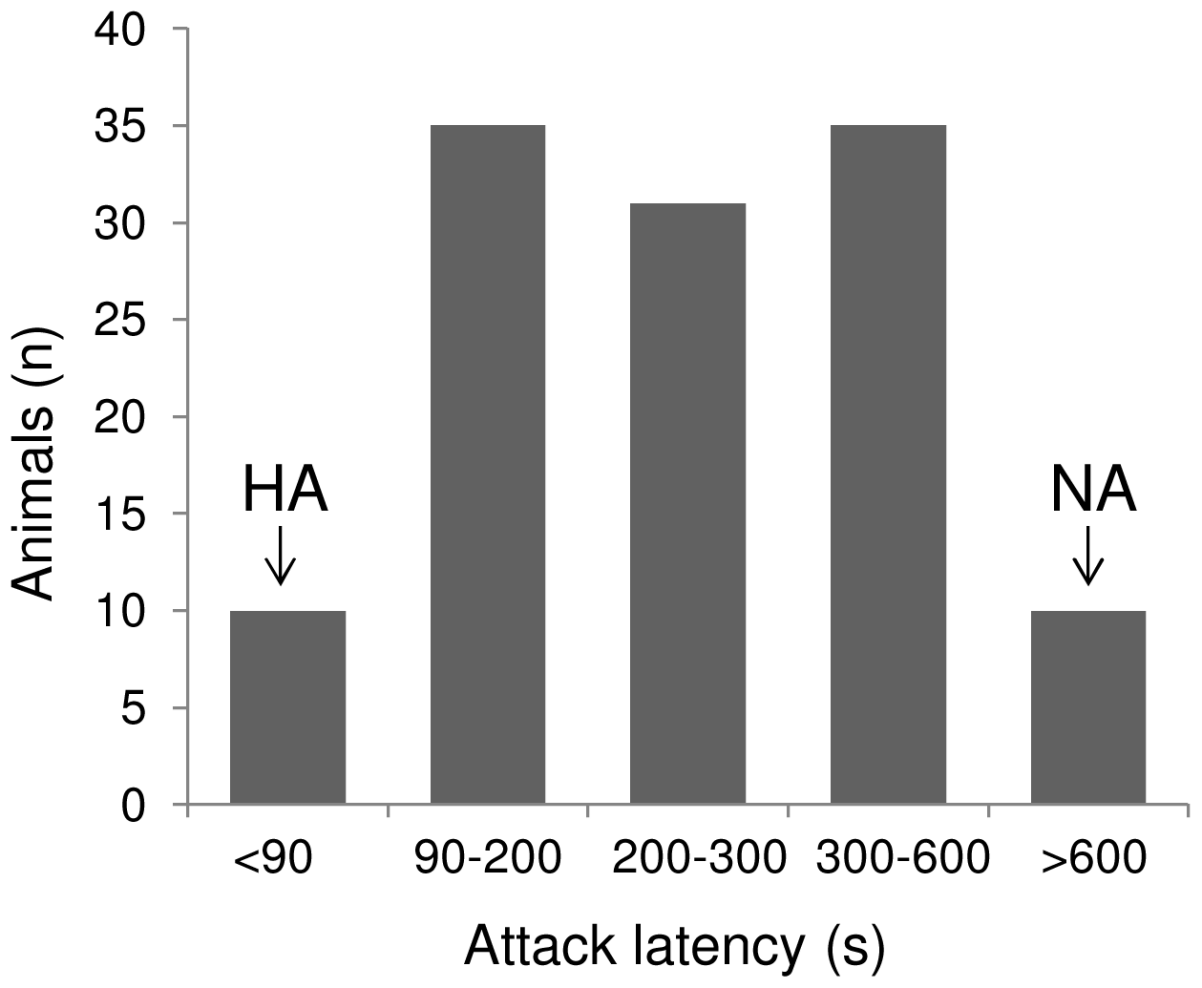
Individual variability in aggressiveness. Distribution of the individual variation in attack latency time towards an unfamiliar intruder within the population (n = 121) of Wild-type rats. On the x-axis the average (3 tests) attack latency to first attack the intruder rat is categorized. On the y-axis the number of rats in each category is presented. Only rats classified as high-aggressive (HA, attack latency <90 s) and non-aggressive (NA, no overt aggression at all during the 10 min-duration of the tests) were subsequently used for the experimental procedures.

All tests lasted 10 min and the latency to the first attack towards the intruder (in s) was measured. The attack latency (average of 3 tests) was used as an index of individual aggressive behavior. As commonly seen in this rat strain [14], individual male resident rats differed widely in their level of aggression towards unfamiliar intruder males (Figure 1). The ten most aggressive rats (average attack latency = 75±4 s; average number of attacks = 7.4±0.6) were selected and classified as high-aggressive (HA) rats (Figure 1). Ten rats showed no overt aggression at all towards the intruder during the 600-s confrontations and were selected and classified as non-aggressive (NA) rats (Figure 1). HA and NA rats were then used for the following experimental procedures.

### Surgery: Radiotransmitter Implantation

Following the preliminary behavioral testing, HA and NA rats were anesthetized with tiletamine hydrochloride+zolazepam hydrochloride (Zoletil 200 mg/kg, s.c.) Radiotelemetric transmitters (TA11CTA-F40, Data Sciences International, St. Paul, MN) for recording electrocardiogram (ECG), core body temperature (T, °C) and locomotor activity (LOC, expressed as counts/minute, cpm) were implanted according to a procedure described by Sgoifo and colleagues [25]. The transmitter body was placed in the abdominal cavity; one electrode was fixed to the dorsal surface of the xyphoid process and another electrode was placed in the anterior mediastinum close to the right atrium. Such electrode location guarantees high-quality ECG recordings, even during vigorous physical activity. Immediately after surgery, rats were individually housed, injected for 2 days with gentamicin sulfate (Aagent, Fatro, 0.2 ml/kg, s.c.) and allowed 10 days of recovery before the start of experimental recordings.

### Experimental Protocol and Radiotelemetric Recordings

Following recovery from surgery, animals were left undisturbed in their home cages for 6 days for collection of daily rhythms of heart rate (HR, bpm), T and LOC. Subsequently, rats were submitted on different days to: i) pharmacological autonomic blockade (day 1), ii) restraint test (day 2), iii) psychosocial stress test (day 3), and iv) β-adrenoceptor pharmacological stimulation (day 4). These tests (described below) were carried between 10∶00 and 14∶00 (i.e. the dark phase of the light/dark cycle). ECG waves (sampling frequency 1000 Hz), T and LOC signals (sampling frequency 256 Hz) were picked up by a radiotelemetry receiver (RPC-1) and recorded via ART-Silver 1.10 data acquisition system (Data Sciences Int., St. Paul, MN, USA).

### Baseline Daily Rhythms

ECG, T and LOC were sampled around-the-clock for 2 minutes every hour over a period of 6 days for collection of baseline daily rhythms. Each day, separate estimates of HR, HRV indexes (see section ‘Quantification of HRV’ for details), T and LOC were generated as average values of 12 h-light and 12 h-dark phases. These parameters were then further averaged as means of the 6 days of the light and dark phases.

### Pharmacological Autonomic Blockade

The muscarinic receptor antagonist methylscopolamine (0.05 mg/kg, Sigma, St Louis, MO, USA) and the β1-adrenergic receptor antagonist atenolol (2 mg/kg; Sigma, St Louis, MO, USA) were injected s.c. to block vagal and sympathetic influences to the heart in HA and NA rats. After baseline ECG recording (30 min), methylscopolamine was injected to evaluate the relative contribution of the vagal control over HR (vagal modulation), which was calculated as the difference between HR under vagal blockade with methylscopolamine (15-min mean) and resting HR (30-min mean). Fifteen min after methylscopolamine injection, atenolol was administered to the same rats to determine intrinsic heart rate (IHR). The doses and time courses of methylscopolamine and atenolol injections were selected on the basis of the available literature data [26]. IHR is established when the cardiac autonomic nervous system is completely blocked, which, in this instance, is supposed to take place approximately 15 min after the sympathetic blocker injection [27] [28].

### Restraint Test

Each animal was introduced for 15 min into a restrainer fitted closely to the body size (wire-mesh tube; inner diameter: 6 cm, length: 20 cm). After the test, animals were returned to their home cages. Continuous ECG, T and LOC recordings were performed in baseline conditions (30 min, prior to the test), during the restraint test (15 min) and throughout the recovery period (45 min).

### Psychosocial Stress Test

A male Wistar rat was transferred to the cage of each experimental rat, with a wire mesh partition separating the two opponents in order to avoid direct physical contact. During this phase (15 min), HA and NA resident rats were in constant sensory contact with the intruder. As demonstrated in a previous study, the mere presence of a potentially antagonist individual in the home cage elicits a strong psychosocial stress response (elevation of plasma corticosterone levels) in both aggressive and nonaggressive resident rats [29]. Moreover, cardiac autonomic reactivity during psychosocial stress test (adverse social contact without overt fighting) in intruder rats was shown to be as large as during an actual fight experience [30]. Continuous ECG, T and LOC recordings were performed in baseline conditions (30 min, prior to the test), during the test (15 min) and throughout the recovery period (45 min).

### β-adrenoceptor Pharmacological Stimulation

After baseline ECG recording (30 min), the β-adrenoceptor agonist isoproterenol (0.02 mg/kg, Sigma, St Louis, MO, USA) was injected s.c. to HA and NA rats. The dose of isoproterenol injection was selected on the basis of the available literature data [31]. ECG recordings were performed for 15 min after isoproterenol administration to evaluate the chronotropic and proarrhythmic effects of β-adrenoceptor stimulation.

### Quantification of HRV

HRV analysis was conducted on multiple segments of stable, continuous ECG signals recorded during: i) baseline daily rhythms (segment duration: 2 min) and ii) restraint test, psychosocial stress test and pharmacological manipulations (segment duration: 5 min). Initially, each raw ECG signal was manually inspected to ensure that all R-waves were correctly detected. We then calculated HR by plotting the number of R waves per unit time (reported in beats per minute; bpm). Subsequently, we quantified time- and frequency-domain parameters of HRV. In the time-domain, we obtained the square root of the mean squared differences of successive RR intervals (RMSSD, ms), which quantifies short-term, high-frequency variations of RR interval and therefore estimates the activity of the parasympathetic nervous system [32]. For spectral (frequency-domain) analysis of HRV, the power spectrum was obtained with a fast Fourier transform-based method (Welch’s periodogram: 256 points, 50% overlap, and Hamming window). We considered the total power of the spectrum (ms^2^) and the power of the low frequency (LF; 0.2–0.75 Hz) and high frequency (HF; 0.75–2.5 Hz) bands in absolute values (ms^2^). The power of LF band is a non-specific index as it contains contributions of both the sympathetic and parasympathetic influences [33]; the power of HF band is due to the activity of the parasympathetic nervous system and includes respiration-linked oscillations of HR [34]. The low frequency/high frequency ratio (LF/HF) estimates the fractional distribution of power and is taken as a synthetic measure of sympathovagal balance [35]. Those parts of ECG recordings which were non-stationary and/or exhibited recording artifacts were excluded from the analysis in accordance to an automatic test checking stationarity of the mean and variance of HR [36] [35].

### Quantification of Arrhythmic Events

The occurrence of arrhythmic events in baseline conditions, under stressful stimuli and following β-adrenoceptor pharmacological stimulation was determined and quantified off-line based on the Lambeth Conventions for the study of experimental arrhythmias [37]. We determined and quantified the total number of arrhythmic events and the separate occurrence of supraventricular and ventricular ectopic beats, either as isolated or grouped events.

### Data Analyses

Data are presented as means ± standard error of the mean (SEM) for all analyses, tables and figures.

Two-way ANOVA for repeated measures was applied for data obtained from: i) baseline daily rhythms, with group as between-subject factor (2 levels: HA and NA) and time as within-subject factor (2 levels: light and dark phases); ii) restraint and psychosocial stress, with group as between-subject factor (2 levels: HA and NA) and time as within-subject factor (5 levels: baseline; test; recovery 1, 2, and 3). Follow-up analyses were conducted using Student’s “t” tests, with a Bonferroni correction for multiple comparisons for each outcome variable separately. A priori Student’s “t”-tests, after controlling for homogeneity of variance via Levene test, were applied for comparisons between HA and NA rats on the occurrence of arrhythmic events and pharmacological manipulations. Statistical significance was set at p<0.05.

## Results

### Daily Rhythms of Radiotelemetric Parameters

The daily rhythms of HR, HRV, T and LOC under resting conditions are presented in Table 1.

**Table 1:** Daily rhythms of radiotelemetric and HRV parameters.

		Light	Dark
HR (bpm)	HA	334±4	371±6
	NA	329±10	365±9
RMSSD (ms)	HA	2.34±0.19*	1.91±0.14^#^
	NA	2.94±0.20	2.45±0.12
Total Power (ms^2^)	HA	53.0±5.7*	36.8±3.5*
	NA	66.7±4.9	56.3±7.9
LF Power (ms^2^)	HA	1.91±0.33*	1.31±0.25*
	NA	3.27±0.49	2.38±0.31
HF Power (ms^2^)	HA	2.27±0.29	1.49±0.19*
	NA	3.22±0.42	2.24±0.27
LF/HF	HA	0.84±0.09	0.88±0.08
	NA	1.01±0.08	1.06±0.09
T (°C)	HA	37.7±0.1	38.0±0.1
	NA	37.6±0.1	37.9±0.1
LOC (cpm)	HA	2.7±0.2	3.8±0.2
	NA	2.9±0.3	4.2±0.2

For the 12 h-light and 12 h-dark phases, values are reported as means ± SEM of data obtained by averaging multiple 2-min segments acquired every hour over a period of 6 days, in high-aggressive (HA, n = 10) and non-aggressive (NA, n = 10) rats. Abbreviations: HRV = heart rate variability; HR = heart rate; RMSSD = square root of the mean squared differences of successive RR intervals; LF = low-frequency; HF = high-frequency; T = body temperature; LOC = locomotor activity. * and ^#^ indicate a significant difference between HA and NA rats (p<0.05 and p<0.01, respectively).

Two-way ANOVA yielded main effects of: i) time for HR values (F = 166.7, p<0.01), RMSSD values (F = 35.4, p<0.01), total spectral power (F = 28.7, p<0.01), spectral power in LF and HF bands (LF = , 35.4, p<0.01; HF = 39.4, p<0.01), T (F = 161.9, p<0.01) and LOC values (F = 41.8, p<0.01); and ii) group for RMSSD values (F = 6.4, p<0.05), total spectral power (F = 7.2, p<0.05) and spectral power in LF band (F = 6.4, p<0.05).

HA and NA rats had similar HR values in both phases of the light-dark cycle (Table 1). However, time-domain analysis of HRV indicated that HA rats had significantly lower values of RMMSD than NA rats during both the light (t = −2.14, p<0.05) and the dark (t = −2.86, p<0.01) phases (Table 1). Frequency-domain analysis of HRV revealed that: i) total spectral power was significantly lower in HA rats than NA rats during the light (t = −2.56, p<0.05) and the dark (t = −2.51, p<0.05) phases (Table 1); ii) spectral power in LF band resulted significantly lower in HA rats compared to NA rats in both phases (light: t = −2.2, p<0.05; dark: t = −2.59, p<0.05) and iii) spectral power in HF band was also lower in HA rats, although statistical significance was reached only during the dark phase (t = −2.25, p<0.05) (Table 1). No differences between groups were observed for LF/HF values (Table 1), in accordance with the absence of significant differences in HR.

HA and NA rats had similar T and LOC values during both the light and the dark phases (Table 1).

### Pharmacological Autonomic Blockade

**Figure 2:**
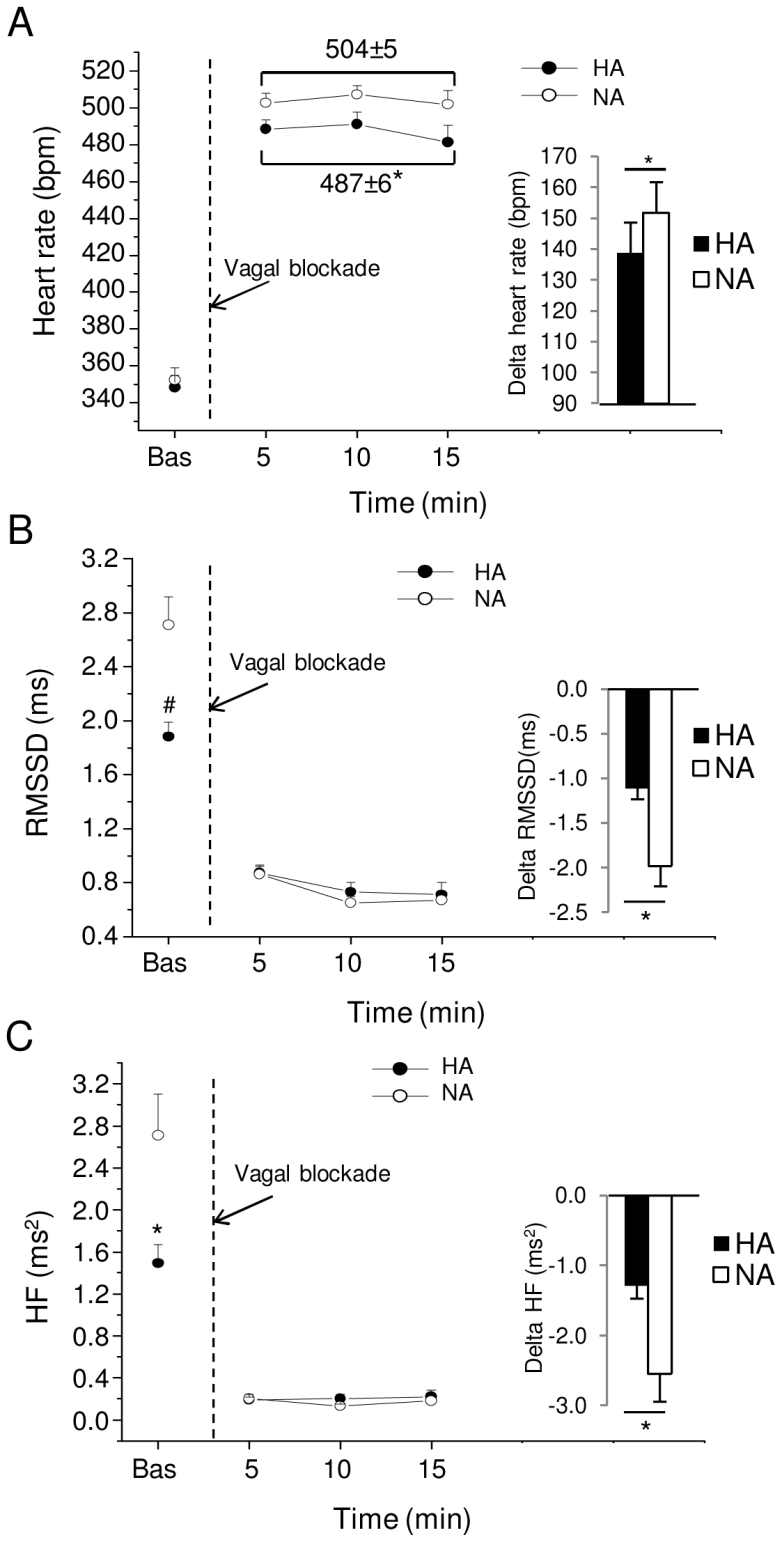
Effects of vagal blockade. Time course of changes in heart rate (panel A), RMSSD values (panel B) and high-frequency (HF) spectral power (panel C), in baseline conditions (bas) and after vagal blockade with metylscopolamine, in high-aggressive (HA, n = 10) and non-aggressive (NA, n = 10) rats. Baseline reference value (bas) is the mean value of the six 5-min time points in resting conditions. Values are expressed as mean ± SEM. Inner graphs in (A), (B) and (C) represent delta values, which were calculated as the difference between 15-min mean values under vagal blockade and basal values obtained during pre-injection recordings (30 min). * and ^#^ indicate a significant difference between HA and NA rats (p<0.05 and p<0.01, respectively).

During baseline, pre-injection ECG recordings HA and NA rats had similar HR (Figure 2A). However, HA rats had significantly lower values of RMSSD (t = −3.42, p<0.01) and spectral power in HF band (t = −2.72, p<0.05) than NA rats (Figure 2B, C). No differences between groups were observed for LF/HF values (HA = 1.05±0.14 vs. NA = 1.13±0.12), in accordance with the absence of significant differences in baseline HR.

Injection of the muscarinic antagonist methylscopolamine provoked a rapid rise in HR, with the magnitude of this HR increment (vagal modulation) being smaller in HA than NA rats (t = −2.1, p<0.05) (Figure 2A). As expected, muscarinic receptor blockade provoked a marked reduction of RMSSD values and HF spectral power, with the magnitude of this decrement being smaller in HA than NA rats (RMSSD: t = −3.4, p<0.05; HF: t = −2.8, p<0.05) (Figure 2B, C).

IHR, evaluated after blockade of autonomic neural modulation with methylscopolamine and atenolol, was similar between the two groups (HA = 362±6 bpm vs. NA = 374±7 bpm).

### Restraint Test

**Figure 3:**
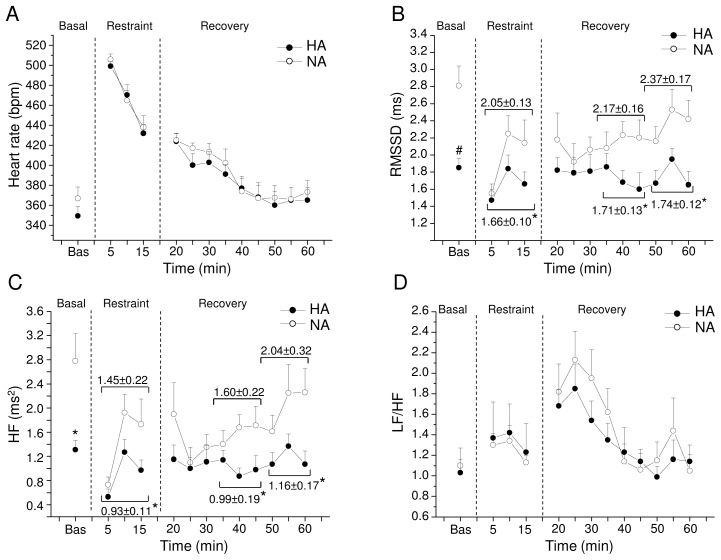
Cardiac autonomic response to the restrain test. Time course of changes in heart rate (panel A), RMSSD values (panel B), high-frequency (HF) spectral power (panel C), and LF to HF ratio (panel D) in baseline conditions (bas), during the restraint and the recovery phase, in high-aggressive (HA, n = 10) and non-aggressive (NA, n = 10) rats. Baseline reference value (bas) is the mean value of the six 5-min time points in resting conditions. Values are expressed as mean ± SEM. * and ^#^ indicate a significant difference between HA and NA rats (p<0.05 and p<0.01, respectively).

Cardiac autonomic responses to the restraint test are depicted in Figure 3 and detailed in Table 2.

**Table 2:** Radiotelemetric and HRV parameters during the restraint test.

		Baseline	Restraint	Recovery (min 0–15)	Recovery (min 15–30)	Recovery (min 30–45)
HR (bpm)	HA	349±9	467±6	409±9	379±8	365±12
	NA	367±11	470±7	418±6	381±13	368±9
RMSSD (ms)	HA	1.85±0.10^#^	1.66±0.10*	1.81±0.16	1.71±0.13*	1.74±0.12*
	NA	2.81±0.22	2.05±0.13	2.05±0.19	2.17±0.16	2.37±0.17
Total Power (ms^2^)	HA	68.9±9.8	23.6±3.2	31.6±3.9	35.9±4.9	43.8±6.1
	NA	73.9±11.5	27.3±3.3	38.7±5.7	33.1±3.2	63.1±13
LF Power (ms^2^)	HA	1.38±0.29*	1.36±0.15	1.71±0.26	1.24±0.20	1.14±0.15^#^
	NA	2.95±0.61	1.83±0.29	2.53±0.36	1.80±0.27	2.12±0.25
HF Power (ms^2^)	HA	1.31±0.15*	0.93±0.11*	1.09±0.20	0.99±0.13*	1.16±0.17*
	NA	2.78±0.43	1.45±0.22	1.45±0.28	1.60±0.22	2.04±0.32
LF/HF	HA	1.03±0.13	1.35±0.32	1.68±0.15	1.24±0.12	0.98±0.13
	NA	1.10±0.17	1.26±0.08	1.92±0.23	1.22±0.16	1.22±0.20
T (°C)	HA	37.8±0.1	38.1±0.1	38.7±0.1	38.4±0.1	38.1±0.1
	NA	37.9±0.1	38.3±0.1	38.8±0.1	38.5±0.1	38.2±0.2
LOC (cpm)	HA	2.4±0.7	4.0±0.6	10.0±1.4	2.4±0.6	2.7±0.7
	NA	3.4±0.9	4.6±0.6	12.6±1.1	4.1±0.9	3.5±1.1

Values are reported as means ± SEM of data obtained by averaging multiple 5-min segments acquired in baseline conditions (30 min), during the restraint (15 min) and the recovery phase (45 min), in high-aggressive (HA, n = 10) and non-aggressive (NA, n = 10) rats. Abbreviations: HRV = heart rate variability; HR = heart rate; RMSSD = square root of the mean squared differences of successive RR intervals; LF = low-frequency; HF = high-frequency; T = body temperature; LOC = locomotor activity. * and ^#^ indicate a significant difference between HA and NA rats (p<0.05 and p<0.01, respectively).

Two-way ANOVA yielded main effects of: i) time for HR values (F = 14.4, p<0.01), total spectral power (F = 5.3, p<0.05) and spectral power in LF band (F = 7.5, p<0.01), and ii) group for RMSSD values (F = 6.1, p<0.05) and spectral power in HF band (F = 6.4, p<0.05).

Before the test, baseline HR was similar between the two groups (Figure 3A and Table 2). However, RMSSD values were significantly lower in HA than NA rats (t = −3.7, p<0.01) (Figure 3B and Table 2). Similarly, spectral power in LF and HF bands was significantly lower in HA than NA rats (LF: t = −2.1, p<0.05; HF: t = −3.0, p<0.05) (Figure 3C and Table 2). During the restraint phase, HR was similar between the two groups (Figure 3A and Table 2). However, in the same period RMSSD values and spectral power in HF band were significantly lower in HA than NA rats (RMSSD: t = −2.1 p<0.05; HF: t = −2.1 p<0.05) (Figure 3B, C and Table 2). No differences between groups were observed for LF/HF values (Table 2), in accordance with the absence of significant differences in HR. During the recovery phase, HR values were similar between HA and NA rats (Figure 3A and Table 2). RMSSD values and spectral power in HF band were significantly lower in HA than NA rats during the second (RMSSD: t = −2.1, p<0,05; HF: t = −2.3, p<0.05) and third (RMSSD: t = −2.8, p<0,05; HF: t = −2.3, p<0.05) 15-min recovery phase (Figure 3B, C and Table 2).

**Figure 4:**
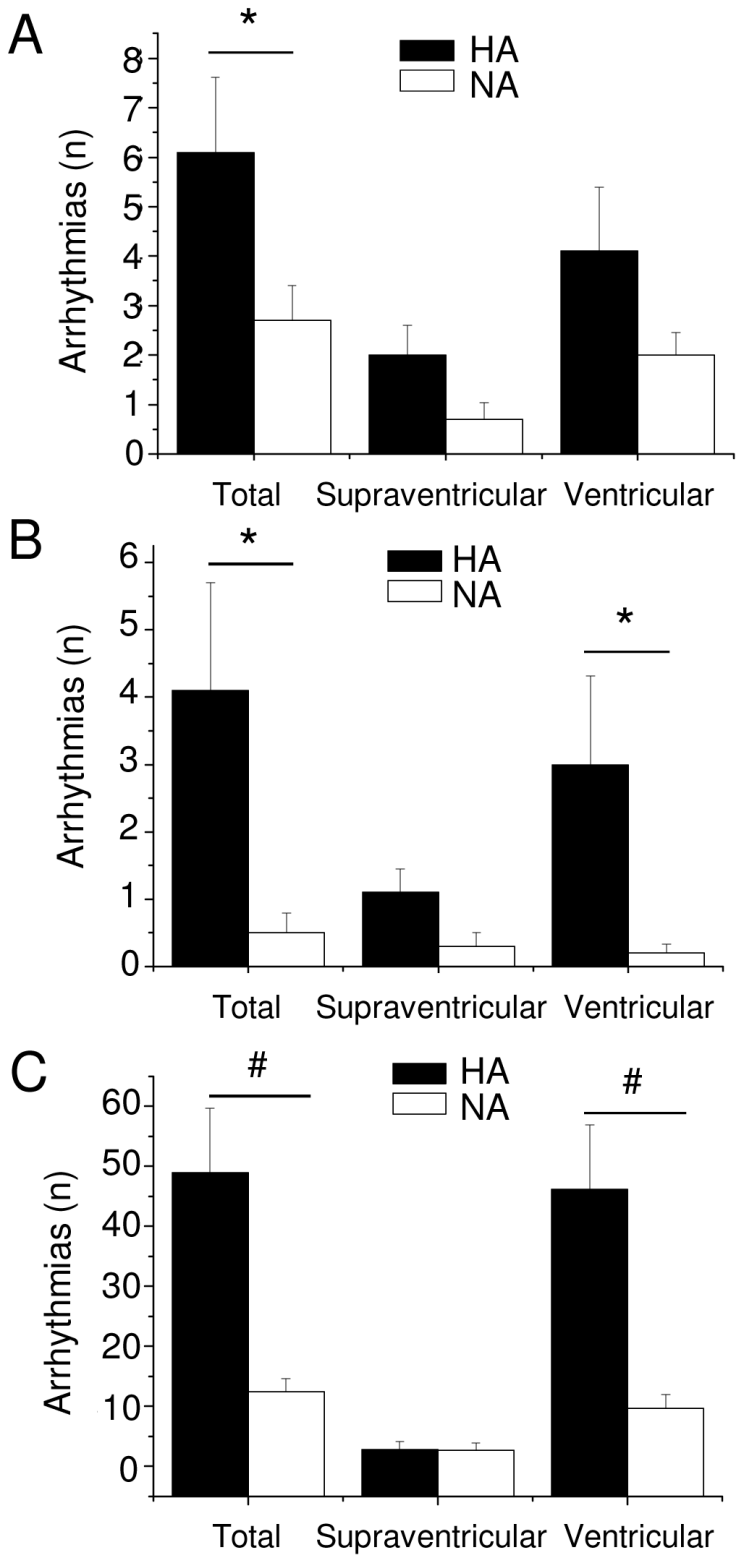
Susceptibility to cardiac arrhythmias. Incidence of arrhythmias during the restraint (panel A), during the psychosocial stress (panel B) and following β-adrenoceptor pharmacological stimulation (panel C), in high-aggressive (HA, n = 10) and non-aggressive (NA, n = 10) rats. Values are reported as mean ± SEM of number of events (n) per 15-min recording period. * and ^#^ indicate a significant difference between HA and NA rats (p<0.05 and p<0.01, respectively).

In addition, during the restraint test HA rats displayed a significantly higher incidence of arrhythmic events compared to NA rats (t = 2.1, p<0.05) (Figure 4A). No differences between groups were found when ventricular and supraventricular arrhythmic events were considered separately (Figure 4A).

No differences between the two groups were observed for T and LOC values, neither during baseline recordings nor during and after the test (Table 2).

### Psychosocial Stress Test

**Figure 5:**
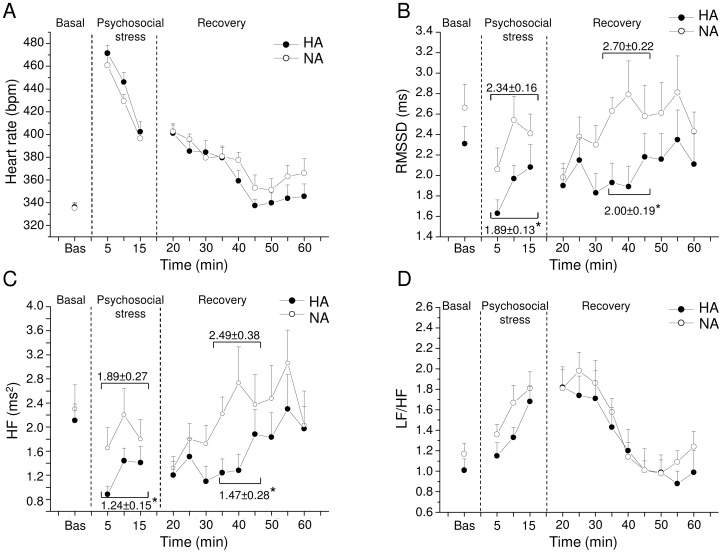
Cardiac autonomic response to the psychosocial stress test. Time course of changes in heart rate (panel A), RMSSD values (panel B), high-frequency (HF) spectral power (panel C), and LF to HF ratio (panel D) in baseline conditions (bas), during the psychosocial stress and the recovery phase, in high-aggressive (HA, n = 10) and non-aggressive (NA, n = 10) rats. Baseline reference value (bas) is the mean value of the six 5-min time points in resting conditions. Values are expressed as mean ± SEM. * indicates a significant difference between HA and NA rats (p<0.05).

Cardiac autonomic responses to the psychosocial stress test are depicted in Figure 5 and detailed in Table 3.

**Table 3:** Radiotelemetric and HRV parameters during the psychosocial stress test.

		Baseline	Psychosocial stress	Recovery(min 0–15)	Recovery(min 15–30)	Recovery (min 30–45)
HR (bpm)	HA	336±4	440±7	390±8	361±8	343±9
	NA	335±4	430±7	392±2	367±8	360±9
RMSSD (ms)	HA	2.31±0.17	1.89±0.13*	1.96±0.17	2.00±0.19*	2.21±0.19
	NA	2.66±0.23	2.34±0.16	2.22±0.13	2.70±0.22	2.62±0.25
Total Power (ms^2^)	HA	82.8±8.7	30.6±3.6	35.5±4.4	48.2±7.1	59.7±10.0
	NA	91.0±13.4	42.2±6.0	49.9±7.6	49.7±7.2	60.5±9.0
LF Power (ms^2^)	HA	2.14±0.30	1.73±0.25*	2.14±0.41	1.57±0.31*	1.59±0.31
	NA	2.68±0.41	2.99±0.44	3.04±0.57	3.20±0.59	2.72±0.62
HF Power (ms^2^)	HA	2.11±0.27	1.24±0.15*	1.27±0.21	1.47±0.28*	2.03±0.52
	NA	2.30±0.40	1.89±0.27	1.61±0.19	2.49±0.38	2.52±0.51
LF/HF	HA	1.01±0.11	1.40±0.10	1.69±0.17	1.07±0.17	0.78±0.17
	NA	1.17±0.10	1.58±0.11	1.88±0.15	1.29±0.10	1.08±0.15
T (°C)	HA	37.8±0.1	38.2±0.1	39.0±0.1	38.7±0.1	38.3±0.1
	NA	37.7±0.1	38.3±0.1	39.0±0.1	38.7±0.1	38.2±0.1
LOC (cpm)	HA	1.8±0.4	15.5±3.4	14.4±1.3	5.2±1.2	2.4±0.8
	NA	2.1±0.5	19.0±2.3	16.0±1.8	5.3±1.7	4.6±1.2

Values are reported as means ± SEM of data obtained by averaging multiple 5-min segments acquired in baseline conditions (30 min), during the psychosocial stress (15 min), and the recovery phase (45 min), in high-aggressive (HA, n = 10) and non-aggressive (NA, n = 10) rats. Abbreviations: HRV = heart rate variability; HR = heart rate; RMSSD = square root of the mean squared differences of successive RR intervals; LF = low-frequency; HF = high-frequency; T = body temperature; LOC = locomotor activity. * and ^#^ indicate a significant difference between HA and NA rats (p<0.05 and p<0.01, respectively).

Two-way ANOVA yielded main effects of: i) time for HR values (F = 5.4, p<0.05) and total spectral power (F = 5.9, p<0.05)), and ii) group for RMSSD values (F = 6.1, p<0.05).

During psychosocial stress HR was similar between the two groups (Figure 5A and Table 3). However, in the same period RMSSD values and spectral power in LF and HF bands were significantly lower in HA than NA rats (RMSSD: t = −2.1 p<0.05; LF: t = −2.5 p<0.05; HF: t = −2.1 p<0.05) (Figure 5B, C and Table 3). No differences between groups were observed for LF/HF values (Figure 5D and Table 3), in accordance with the absence of significant differences in HR. During the recovery phase HR values were similar between HA and NA rats (Figure 5A and Table 3), while RMSSD values and spectral power in HF band were significantly lower in HA than NA rats only during the second 15-min recovery phase (RMSSD: t = −2.4, p<0,05; HF: t = −2.2, p<0.05) (Figure 5B, C and Table 3).

In addition, during the psychosocial stress test HA rats displayed a significantly higher incidence of arrhythmias (t = 2.2, p<0.05) (Figure 4B), which was mainly due to a significantly larger incidence of ventricular ectopic events (t = 2.1, p<0.05) compared to NA rats (Figure 4B).

No differences between the two groups were observed for T and LOC values, neither during baseline recordings nor during and after the test (Table 3).

### β-adrenoceptor Pharmacological Stimulation

HA and NA rats had similar HR during baseline, pre-injection ECG recordings (HA = 339±10 bpm vs. NA = 323±7 bpm).

**Figure 6:**
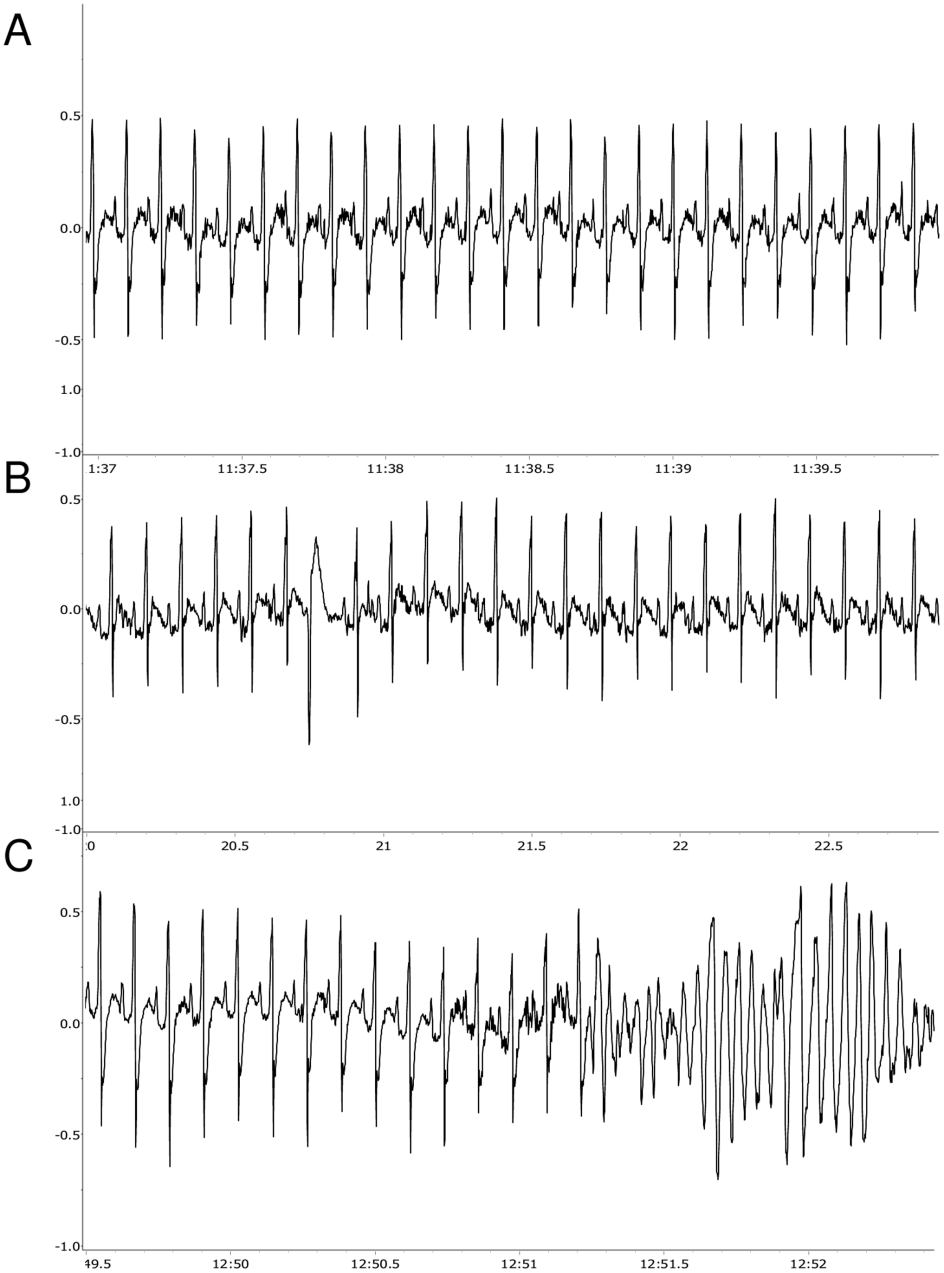
Lethal ventricular tachycardia in a high-aggressive rat. ECG traces belonging to a high-aggressive rat that died following β-adrenoceptor stimulation with isoproterenol. A: normal ECG. B: ECG with an isolated premature ventricular complex. C: ECG before rat’s death with sustained ventricular tachycardia.

After injection of the β-adrenoceptor agonist isoproterenol, HR was similar between the two groups (HA = 488±4 bpm vs. NA = 475±10 bpm). However, following β-adrenoceptor stimulation with isoproterenol, HA rats displayed a dramatically higher incidence of arrhythmias compared to NA rats (t = 3.5, p<0.01) (Figure 4C). The increased arrhythmogenesis in HA rats was exclusively due to a significantly larger incidence of ventricular arrhythmias compared to NA rats (t = 3.5, p<0.01), whereas the occurrence of supraventricular arrhythmias was modest and similar between the two groups (Figure 4C). In addition, after β-adrenoceptor stimulation with isoproterenol, one HA rat displayed sustained ventricular tachycardia (Figure 6) that led to death.

## Discussion

The purpose of this study was to characterize cardiac autonomic activity in Wild-type rats that differed widely in their level of aggressive behavior. To reach this goal, autonomic neural modulation was studied at rest and under different stress conditions by means of HRV analysis, which relies on the principle that the pattern of beat-to-beat control of the sinoatrial (SA) node provides a reflection of cardiac autonomic regulation. We found that high-aggressive rats had: i) lower HRV in resting conditions, ii) lower vagal modulation of heart rate at rest and under stress conditions, and iii) larger incidence of arrhythmias induced by stress or by pharmacological β-adrenergic stimulation compared to non-aggressive rats. These findings are consistent with the view that high levels of aggressive behavior in rats are associated with specific features of cardiac autonomic modulation and increased arrhythmogenic susceptibility that may predict vulnerability to cardiac morbidity and mortality.

The Wild-type Groningen rat is a rodent species that shows a high individual variability in the tendency to behave aggressively or cope (pro)actively in response to stressful stimuli [19]. In our view, the selection of high-aggressive and non-aggressive subjects within this rat strain is a reliable approach for investigating the autonomic correlates of extremes in aggressive behavior [14]. The analysis of HRV is a widely used tool for obtaining detailed information on the relative contribution of sympathetic and vagal regulation of cardiac (re)activity [38]. Although there is an ongoing debate regarding the suitability of using HRV parameters to estimate sympathetic modulation [39], this approach produces reliable measures of vagal tone during both undisturbed resting and stress conditions [34] [39]. Particularly, parasympathetic/vagal modulation is a useful indicator for determining the behavioral and physiological flexibility of an organism and for measuring their ability to adequately respond to stress [40].

Initially, HRV parameters were evaluated during baseline rhythm recordings (6 days) in order to obtain a valid characterization of cardiac autonomic regulation under normal undisturbed resting conditions. Overall, we found that HA rats exhibited a reduced HRV compared to NA counterparts during both the light and dark phases of the circadian rhythm. As suggested by RMSSD and HF power indexes, HA rats were characterized by a lower vagal modulation of heart rate. An important confirmation of a reduced vagal modulation of heart rate in HA rats resulted from the pharmacological challenge with methylscopolamine. The injection of this vagal blocker led to a lower heart rate increase in HA rats than NA rats, confirming a lower relative contribution of the vagal control over resting heart rate in HA animals. The fact that vagal blockade provoked a milder reduction of RMSSD and HF power values in HA than NA rats was due to significant differences in baseline values, which were much lower in HA rats. Despite the difference in cardiac vagal tone, HA and NA rats had similar heart rate values at rest. One possible explanation for this apparent discrepancy may be that in HA rats the reduced cardiac vagal tone was coupled with decreased cardiac sympathetic influence on the SA node compared to NA rats. This is supported by the fact that the two groups did not differ for LF to HF ratio (index of sympathovagal balance), suggesting that the regulatory influences of the vagal and sympathetic components on cardiac pacemaker activity were similarly balanced in the two groups, leading to similar heart rate values. In addition, intrinsic heart rate, measured after double autonomic blockade and therefore free from autonomic modulation, was similar between the two groups, suggesting that high-aggressive and non-aggressive rats had similar intrinsic automaticity of the SA node.

Subsequently, we tested the two groups under stress conditions. We found that HA and NA rats showed similar heart rate and body temperature responses to the restraint and psychosocial stress tests. This is an interesting and unexpected phenomenon, as previous studies have clearly demonstrated that high-aggressive Wild-type rats have a larger sympatho-adrenomedullary reactivity (plasma noradrenaline and adrenaline levels) to acute stress challenges compared to less aggressive rats [21] [22]. Therefore, one would expect larger tachycardic and hyperthermic stress responses in high-aggressive rats. Given that the autonomic physiological target-organ responses (i.e., heart rate, body temperature) were instead not different between HA and NA animals, our first hypothesis was that vagal (re)activity was also higher in HA animals. This, however, was not the case, as the vagal indexes of RMSSD and HF power indicated that HA rats had lower cardiac vagal modulation compared to NA rats. Based on these observations, we hypothesize that adrenergic receptors in the SA node and skin vessels are desensitized/downregulated in HA animals. This adrenergic receptor remodeling would also explain why the influences of sympathetic and vagal modulations on the pacemaker region were similarly balanced in the two groups (LF to HF ratio index).

Interestingly, during the restraint and the psychosocial stress tests, HA rats showed a larger susceptibility to tachyarrhythmias compared to NA counterparts. Arrhythmogenesis was clearly stress-induced, as no arrhythmic events were noted during pre-stress recordings. Alterations in cardiac autonomic modulation are thought to exert a potent influence on arrhythmogenesis (for a review see [41]). Increases in sympathetic nerve activity, through the influence of noradrenaline on beta-adrenergic receptors, participate in the genesis of ventricular tachyarrhythmias. On the other hand, decreases in vagal tone leave the heart exposed to unopposed stimulation by the sympathetic nervous system, and consequently vulnerable to ventricular arrhythmia and sudden death [42]. In our study, HA rats exhibited a lower vagal antagonism during stress compared to NA rats, supporting the hypothesis that vagal tone impairment may represent a potential triggering mechanism for the increased arrhythmogenesis observed during stress in HA rats. However, we cannot exclude that the larger incidence of arrhythmias induced by stress in HA rats was due to a combination of a vagal dysfunction and the effects of elevated levels of circulating catecholamines [21] [22] on the electrical stability of the ventricular myocardium.

The arrhythmogenic susceptibility in HA rats was even more dramatically evident after β-adrenergic pharmacological stimulation with isoproterenol. In one HA rat the injection of the β-receptor agonist provoked sustained ventricular tachycardia that led to death. It is interesting to note that arrhythmias were almost exclusively of ventricular origin, whereas supraventricular arrhythmias were just occasionally noted. In addition, heart rate response to β-adrenergic pharmacological stimulation was similar between the two groups. These findings suggest that HA rats might have a higher sensitivity/density of ventricular β-adrenergic receptors compared to NA rats. On the other hand, we speculate that HA rats might be characterized by changes in the electrical properties of the ventricular myocardium (e.g., altered refractoriness and/or conduction [43]) that could potentially be pro-arrhythmic under conditions of strong β-adrenergic stimulation.

### Conclusion and Perspectives

In humans, there is strong prospective evidence that reduced heart rate variability and suppressed vagal control over cardiac function contribute to arrhythmogenesis and predict premature cardiac morbidity and mortality [11] [44] [10]. In this study on Wild-type rats we document an association among high levels of aggressive behavior, specific patterns of cardiac autonomic neural modulation, and increased arrhythmogenic susceptibility that may predict vulnerability to cardiac morbidity and mortality. We demonstrate that high-aggressive Wild-type rats are characterized by lower vagal activity at rest and poorer vagal antagonism during stress, and by a much larger susceptibility to stress- and pharmacologically-induced arrhythmias. These results highlight the relevance of this rodent model for the study of the link between aggressive behavior and cardiac disease vulnerability. Further, these findings provide a strong foundation for future mechanistic experiments that will determine: i) the central neural determinants of the described vagal control impairment, and ii) the cellular and subcellular bases of the reported arrhythmogenesis.
